# Efficacy of epicutaneous immunotherapy (EPIT) in a new model of peanut-induced eosinophilic esophagitis (EoE) and allergic enterpathy (AE)

**DOI:** 10.1186/2045-7022-1-S1-O50

**Published:** 2011-08-12

**Authors:** Lucie Mondulet, Vincent Dioszeghy, Véronique Dhelft, Mélanie Ligouis, Emilie Puteaux, Thibaut Larcher, Yan Cherel, Christophe Dupont, Pierre-Henri Benhamou

**Affiliations:** 1 DBV Technologies, Paris, France; 2 APEX, INRA, Nantes, France; 3 Hopital Saint Vincent de Paul, Paris, France

## Background

Eosinophilia is often linked to allergic gastrointestinal disorders linked to food allergy. EPIT using Viaskin® device has been described as a therapeutic method in food allergy. We developed a model of mice sensitized to peanut, exhibiting EoE and AE after exclusive feeding with peanut protein extracts (PPE). This study was conducted in order to evaluate the efficacy of EPIT.

## Methods

After oral sensitization with PPE and cholera toxin, 30 BALB/c mice were treated weekly during 8 weeks by PPE skin applications (EPIT), 20 mice were not treated (Sham) and 10 mice constituted the control group (C). Mice were then exclusively fed with PPE. Specific IgE, IgG1 and IgG2a were monitored during immunotherapy. Esophageal and jejunal samples were taken for histological analyses.

## Results

sIgE increased after oral sensitization, respectively 0.207±0.03 and 0.214±0.04 μg/ml, in EPIT and Sham, with undetectable values in C. Following EPIT, sIgE decreased and sIgG2a increased, respectively 0.139±0.01 vs 0.166±0.01 μg/ml (EPIT vs Sham, p<0.05) and 14.96±0.60 vs 4.73±1.75 μg/ml (p<0.05). Esophageal eosinophilic infiltration (measured in 6 high power fields) was higher in Sham, 136±32, than in EPIT, 50±12 (p<0.05) and C, 7±3 cells/mm2 (p<0.01). Esophagus mucosa thickness was increased in Sham compared to EPIT and C (p<0.001). Sham group exhibited higher mRNA levels of cytokines than EPIT: eotaxin (p<0.05), IL-5 (p<0.05), IL-13 (p<0.05). The mRNA levels of these cytokines in EPIT were similar to C. The expression of Foxp3 mRNA increased significantky after EPIT compared with Sham and C (p<0.05). The jejunal villus/crypt ratio was lower in Sham than in EPIT and C, respectively 1.6±0.1 vs 2.3±0.2 (p<0.01) and 2.4±0.1 (p<0.001). Eosinophilic infiltration in jejunum was increased in Sham compared to EPIT (p<0.01) and C (p<0.001).

## Conclusion

EPIT is effective in preventing EoE and AE induced by oral challenge in mice sensitized to peanut.

